# Reverse T_3_ in patients with hypothyroidism on different thyroid hormone replacement

**DOI:** 10.1371/journal.pone.0325046

**Published:** 2025-06-09

**Authors:** Julian B. Wilson, Thanh D. Hoang, Martin L. Lee, Ma’ayan Epstein, Theodore C. Friedman

**Affiliations:** 1 Division of Endocrinology, Metabolism and Molecular Medicine, Department of Internal Medicine, Charles R. Drew University of Medicine and Science, Los Angeles, California, United States of America; 2 Endocrinology Division, Walter Reed National Military Medical Center, Bethesda, Maryland, United States of America; 3 UCLA Fielding School of Public Health, Los Angeles, California, United States of America; 4 David Geffen School of Medicine at University of California, Los Angeles, California, United States of America; Tribhuvan University Institute of Medicine, NEPAL

## Abstract

**Background:**

Reverse T_3_ (rT_3_) is a biologically inactive form of T_3_ (triiodothyronine), a thyroid hormone, that is created by peripheral 5 deiodination of T_4_ (thyroxine) by type 1 and type 3 deiodinase enzymes (D1 and D3 respectively) and may block T_3_ binding to the thyroid hormone receptor. Approximately 15% of patients on L-T_4_ replacement therapy with a normalized thyroid-stimulating hormone (TSH) report experience continued fatigue and other hypothyroid symptoms; therefore, efforts are needed to understand why this occurs and how it can be corrected. Decades ago, endocrinologists realized that in patients with severe illnesses, rT_3_ is typically high and T_3_ is typically low; this was termed “euthyroid sick syndrome”. More recently, functional medicine and other doctors, have argued that high rT_3_ is detrimental and can block T_3_ from binding to the thyroid hormone receptor. Due to the lack of peer-reviewed publications on this topic, functional medicine doctors continue to rely heavily on rT_3_ levels to treat patients that may have no other laboratory findings of hypothyroidism and often prescribe L-T_3_-only preparations to patients in an effort to lower rT_3_.

**Methods:**

The initial rT_3_ measurements done by liquid chromatography/tandem mass spectrometry (LC/MS-MS) were retrospectively analyzed from the initial blood tests in 976 consecutive patients, with symptoms of fatigue and treated for hypothyroidism, in a private Endocrinology practice. TSH, free T_3_ and free T_4_ were measured by electrochemiluminescence immunoassay (ECLIA). The upper limit of normal rT_3_ (24.1 ng/dL) was used as a cut-off for results above the normal range.

**Results:**

The number of patients with rT_3_ levels above normal range varied significantly with the type of thyroid hormone replacement prescribed. The highest rate of an elevated rT_3_ was 20.9% (29/139) in patients taking T_4_ alone. Nine% (31/345) of patients not taking thyroid hormone replacement had elevated rT_3_. Patients on all types of L-T_4_ treatment had higher rT_3_ levels than those not on L-T_4_ treatment (p < 0.00001) and they also had a higher percentage of rT_3_ levels above the cutoff of 24.1 ng/dL (p < 0.00001). Linear regression analysis showed rT_3_ levels correlated with free T_4_ and free T_3_ levels and inversely with log TSH levels.

**Conclusions:**

This study found elevated rT_3_ levels in patients with symptoms of fatigue on various thyroid hormone replacements with the highest levels of rT_3_ in those taking L-T_4_ replacement alone and the lowest levels of rT_3_ in those on preparations that contained L-T_3_ alone.

## 1. Introduction

Under physiological conditions, T_4_ (thyroxine) is primarily monodeiodoniated to T_3_ (3,3´,5-triiodo-L-thyronine) or reverse T_3_ (rT_3_; 3,3´,5´-triiodo-L-thyronine), depending on the energy or T_3_ needs of the body [1]. T_3_ is considered the active hormone because its affinity for thyroid hormone receptors is 15 times higher than T_4_, while rT_3_ inhibits the effects of T_3_ and T_4_ without binding to nuclear thyroid hormone receptors [2–5]. The clinical significance of rT_3_ has been debated since the 1970s, when the newly adopted “thyroid function test” allowed endocrinologists to realize that severe illness causes a reduction in T_3_ and an increase in rT_3_ [6]. Endocrinologists termed this “euthyroid sick syndrome” and noted that it was common in many types of chronic diseases, especially in patients hospitalized in intensive care units. Whether this is an adaptive response (to conserve energy) or a pathological response (where illness leads to reduced T_3_ production below that what is needed, thus warranting thyroid hormone treatment) is still the subject of intense debate. Overall, it is viewed that these patients should not be treated with thyroid medication [7–9].

As for the clinical significance of rT_3_ in thyroidal illness, the data is sparse. In 1977, Burman et al. developed one of the first rT_3_ assays and demonstrated that its levels varied significantly based on a person’s thyroid status (normal, hyperthyroid, and hypothyroid), but also based on the dosage of levothyroxine that patients received [10]. In particular, they showed that patients that were hypothyroid and receiving 0.05 mg per day of levothyroxine (suboptimal dosage) had below normal rT_3_ levels, while patients receiving 0.4 mg per day (supraoptimal dosage) had above normal rT_3_ levels, suggesting that knowledge of rT_3_ levels could be useful in the management of hypothyroidism. However, commercial rT_3_ assays were not available and the measurement of rT_3_ was confined to research settings. In 1995, Burmeister et al. published her evaluation of 246 patients whose rT_3_ levels were measured while being treated in a university teaching hospital. She showed rT_3_ levels varied tremendously and judged its measurement to be unreliable in distinguishing between hypothyroid sick patients and the euthyroid sick patients [11]. Even though this work looked at the variance of rT_3_ levels in nonthyroidal illness, many in the field felt that this unreliability extended to thyroid illness, and it is currently viewed that the measurement of rT_3_ is of little clinical use [12,13].

The authors believe this conclusion should be reevaluated for several reasons. Firstly, rT_3_ measurements have recently become more accurate with the wide-spread use of mass-spectrometry in commercial laboratories [2] and are available at both Quest Diagnostics and Labcorp. Since rT_3_ inhibits the action of the biological hormone T_3_ at the T_3_ receptor, knowledge of rT_3_ levels is required to completely understand the effects of thyroid hormone administration [5,14]. Importantly, thyroid medication is the second most prescribed drug in the U.S. [15] and yet as many as 40–50% of patients with these medications do not take them as prescribed [16]. Furthermore, many patients turn to alternative doctors, including functional medicine doctors, for the management of thyroid illness. These doctors often measure rT_3_ and use it to guide patient treatment. These providers have argued that high rT_3_ is detrimental and can block T_3_ from binding to the thyroid hormone receptor. With little peer-reviewed publications [17], these functional medicine doctors rely heavily on rT_3_ levels to treat patients that may have no other laboratory findings of hypothyroidism and often prescribe them L-T_3_-only preparations to try to lower the rT_3_. Studies looking at rT_3_ with valid assays are needed to determine the role of this hormone. Functional medicine doctors have proposed risk factors for elevated rT_3_ levels including stress, depression, pain, inflammation, dieting and iron deficiency [17].

In this paper, we retrospectively analyzed initial rT_3_ measurements from 976 consecutive patients seen by TCF from 2010 to 2021 in a private Endocrinology practice. Six hundred thirty-one patients were on varying types of thyroid hormone replacement and 345 patients were not on any thyroid hormone replacement.

## 2. Methods

### 2.1 Study population and methodology

The rT_3_ measurements were retrospectively analyzed from initial blood tests along with other thyroid function tests from 976 consecutive patients seen by TCF from 2010 to 2021 in a private Endocrinology practice. Three hundred forty-five patients were not on thyroid hormones, 226 were on desiccated thyroid extract (DTE) (Armour thyroid, NP thyroid, Nature-throid and WP thyroid were the most common brands) and not synthetic thyroid hormones, 15 were on desiccated thyroid and L-T_3_, 138 were on desiccated thyroid and L-T_4,_ 7 were on desiccated thyroid, L-T_3_ and L-T_4,_ 23 were on L-T_3_ alone, 139 were on L-T_4_ alone, and 83 were on L-T_3_ and L-T_4_.

All patients had fatigue as one of their main symptoms and none had a severe chronic disease in which they would be considered “sick euthyroid.” All patients had rT_3_, free T_3_, free T_4_, anti-thyroid peroxidase (anti-TPO), and TSH measured, usually in the morning after their visit at either Quest Diagnostics or Labcorp. rT_3_ at both laboratories were done by liquid chromatography/tandem mass spectrometry (LC/MS-MS). JW and ME. performed chart review and were not able to identify free T_3_, free T_4_, anti-TPO, and TSH in several patients during their chart review. The normal range for rT_3_ at Labcorp was 9.1 to 24.1 ng/dL and Quest Diagnostics was 8.0 to 25.0 ng/dL. The cut-off for results above the range used the value of 24.1 ng/dL and these values were determined in euthyroid patients. TSH, free T_3_ and free T_4_ were measured by electrochemiluminescence immunoassay (ECLIA) with a range of 0.45- 4.5 miU/mL, 2.0 to 4.4 pg/mL and 0.82 − 1.77 ng/dL, respectively at Labcorp and 0.45- 4.5 miU/mL, 2.3-4.2 pg/mL and 0.8-1.8 ng/dL at Quest. Anti-TPO was done by chemiluminescense at Esoterix Laboratories (subsidiary of Labcorp) and Quest with a range of < 9.0 IU/mL at both laboratories.

### 2.2 Statistical analyses

Sub-analyses of the pairwise comparisons of the thyroid treatment groups, and significance of anti-TPO status within these groups were performed using Dunn’s test. The Wilcoxon rank-sum test was used to compare rT_3_ levels between all groups on L-T_4_ treatment and all groups not on L-T_4_ treatment. The chi-square test for homogeneity was used to compare the % of patients with rT_3_ above range between all groups on L-T_4_ treatment and all groups not on L-T_4_ treatment. Pearson correlations between rT3, free T3, free T4 and log TSH (to compensate for severe non-normality) levels were calculated and the significance of these (from zero) were determined by the appropriate t-test. The patients with circulating anti-TPO antibodies who had elevated rT_3_ levels were compared to patients without these antibodies or who did not have them tested were compared by the Fisher’s exact test.

### 2.3 IRB approval

The Charles R. Drew University of Medicine and Science (CDU) Institutional Review Board (IRB) approved this retrospective study under Exemption Category # 4 (45CFR46.104, category 4iii). The most recent approval date was January 3, 2024. The data was accessed for research purposes on January 18, 2022, and accessed again on January 1, 2024. The authors had access to information that could identify individual participants during or after data collection, however the CDU IRB approved the use of PHI as involving no more than minimal risk and did not require a waiver of consent.

### 2.4 Patient and public involvement

Patients and the public were not involved in the design or the interpretation of the study although patients in this study have informed the investigators about the importance of measuring rT_3_.

## 3. Results

**Fig 1:**
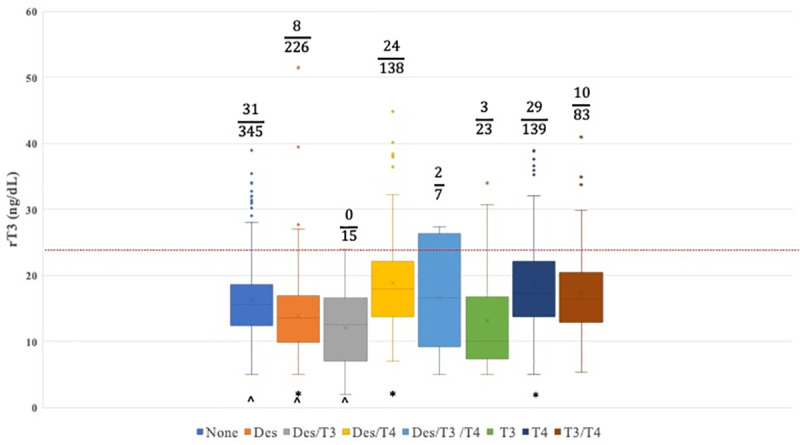
Box and whisker plot of patient rT_3_ based on the type of thyroid hormone replacement. None = Not taking any thyroid hormone replacement; Des = Desiccated thyroid hormone replacement; Des/T_3_ = Desiccated thyroid-T_3_ combination; Des/T_4_ = Desiccated thyroid-T_4_ combination; Des/T_3_/T_4_ = Desiccated thyroid-T_3_-T_4_ combination; T_3_/T_4_ = T_3_-T_4_ combination. * P < 0.05 compared to none. ^ P < 0.05 compared to T_4_.

Table 1 shows the Mean, SD, and N of rT_3_, free T_3_, free T_4_ and TSH in the study population. 810 patients were female and 166 were male. Overall, 107 (11.0) patients had an elevated rT_3_ value. The proportion of patients with above normal rT_3_ values was found to be significantly affected by treatment (Fig 1) with the highest rate of elevated rT_3_ in 20.9% (29/139) of patients taking T_4_ alone. Nine percent (31/345) of patients not taking thyroid hormone replacement had elevated rT_3_ values. In contrast, only 3.5% (8/226) of patients taking desiccated thyroid hormone had above normal rT_3_ values, compared to 12% (10/83) of patients taking a T_3_-T_4_ combination and 17.4% (24/138) of patients taking desiccated thyroid-T_4_ combination. The proportion of patients taking desiccated thyroid hormone replacement with above normal rT_3_ levels was found to be significantly less than all other groups except for patients taking a desiccated-T_3_ combination (Fig 1). Table 2 shows the P-values of pairwise comparisons of the groups using Dunn’s test.

**Table 1:** Mean, SD, N of rT_3_ (ng/dL), Free T_3_ (pg/mL), Free T_4_ (ng/dL) and TSH (µIU/mL).

	rT_3_	FreeT_3_	FreeT_4_	TSH
Mean	16.4	3.2	1.2	2.3
SD	6.6	1.0	0.3	4.3
N	976	452	455	513

**Table 2:** P-values of pairwise comparisons of the groups using Dunn’s test.

	None	Des	Des/T_3_	Des/T_4_	Des/T_3_/T_4_	T_3_	T_4_	T3/T4
**None**	–	0.01	0.22	**0.01**	0.08	0.52	**3x10** ^**-4**^	0.39
**Des**	**0.01**	–	0.46	**6x10** ^**-6**^	**1x10** ^**-3**^	**0.03**	**1x10** ^**-7**^	**5x10** ^**-3**^
**Des/T**_**3**_	0.22	0.46	–	0.08	**0.03**	0.14	**0.05**	0.16
**Des/T**_**4**_	**0.01**	**6x10** ^**-6**^	0.08	–	0.45	0.61	0.46	0.29
**Des/T**_**3**_ **/T**_**4**_	0.08	**1x10** ^**-3**^	**0.03**	0.45	–	0.33	0.63	0.22
**T**_**3**_	0.52	**0.03**	0.14	0.61	0.33	–	0.38	0.90
**T**_**4**_	**3x10** ^**-4**^	**1x10** ^**-7**^	**0.05**	0.46	0.63	0.38	–	0.09
**T3/T4**	0.39	**5x10** ^**-3**^	0.16	0.29	0.22	0.90	0.09	–

None = Not taking any thyroid hormone replacement; Des = Desiccated thyroid hormone replacement; Des/T_3_ = Desiccated thyroid-T_3_ combination; Des/T_4_ = Desiccated thyroid-T_4_ combination; Des/T_3_/T_4_ = Desiccated thyroid-T_3_-T_4_ combination; T_3_/T_4_ = T_3_-T_4_ combination.

Table 3 shows that patients on all types of L-T_4_ treatment had higher mean rT_3_ levels than those not on L-T_4_ treatment (p < 0.0001) and a higher percentage of rT_3_ levels above the cutoff of 24.1 ng/dL (p < 0.0001). Linear regression analysis (Table 4) showed rT_3_ levels strongly correlated with free T_4_ and free T_3_ levels and inversely with log TSH levels.

**Table 3:** Effect of L-T_4_ treatment on rT_3_ levels.

	All groups on L-T_4_ treatment	All groups not on L-T_4_ treatment	p-value
rT_3_ (Mean ± SD)	18.4 ± 7.1	15.2 ± 6.0	<0.00001^*^
% with rT_3_ > 24.1	65/367 (17.7%)	42/609 (6.9%)	p = 1.63 x 10^−7^^

* Wilcoxon rank-sum test, ^^^chi-square test for homogeneity

**Table 4:** Correlations between rT_3_ levels and other hormones.

	FreeT_3_	FreeT_4_	Log TSH
Pearson correlation	0.184	0.624	−0.298
p	0.0001	<0.0001	<0.0001

The presence of anti-TPO antibodies was assessed in 712 of these patients with 212 patients having anti-TPO antibodies above the range and 500 patients not having elevated levels. For the patients not on thyroid hormone replacement, 41 of 345 patients had anti-TPO antibodies and 304 patients did not have antibodies or were not assessed. For the patients on thyroid hormone replacement, 170 of 631 patients had anti-TPO antibodies and 461 patients did not have antibodies or were not assessed. For most thyroid treatment regimens, the proportion of patients with above normal rT_3_ levels did not vary significantly with TPO antibody status, except for patients taking desiccated thyroid hormone replacement. For this group, 0% (0/67) of the patients with circulating anti-TPO antibodies had elevated rT_3_ levels, while 7% (6/88) of patients without these antibodies or who did not have them tested had elevated rT_3_ levels (p = 0.037). For those taking thyroid hormone preparations besides DTE, 20 of 103 had anti-TPO antibodies and 49 of 270 did not have antibodies or were not assessed (p = NS).

## 4. Discussion

The initial rT_3_ measurements were retrospectively analyzed from 976 consecutive patients before management by TCF. Patients with hypothyroidism generally sought out TCF due to dissatisfaction with their current management, including persistent fatigue despite being on what their previous provider considered adequate treatment. We did not track what type of providers treated these patients but posit that it included primary care providers, other Endocrinologists, functional medicine doctors, mid-level health care providers (i.e., physician assistants, nurse practitioners, etc.), and holistic doctors. The high prevalence of patients treated with thyroid preparations other than L-T_4_ reflects the heterogeneity of providers as well as the dissatisfaction with conventional treatment among this group of patients. We found that the proportion of patients with above normal rT_3_ values varied significantly depending on the type of thyroid medication they were taking. This proportion was higher in patients taking preparations containing L-T_4_ but was lower in patients taking desiccated thyroid or L-T_3_ preparations. Groups that took preparations containing desiccated thyroid and/or L-T_3_ with L-T_4_ had a larger proportion of patients with above normal rT_3_ values than groups that took the same preparations without L-T_4_. The group taking L-T_4_ alone had the highest percentage of elevated rT_3_ values at 20.9%. Our results are consistent with previous findings that in short-term settings, L-T_4_ can raise rT_3_ levels [18] and L-T_3_ can lower rT_3_ levels [19], although these effects need to be verified prospectively in larger, newer studies.

Although the majority of patients do have a satisfactory response on L-T_4_ therapy, up to 15% of properly treated hypothyroid patients fail to achieve a sense of well-being on levothyroxine and continue to have hypothyroid symptoms despite normalized thyrotropin levels. The causes of patients’ lack of well-being have been discussed including by the American Thyroid Association Task Force on Thyroid Hormone Replacement [20] and include decreased serum T_3_/T_4_ ratio and alterations in the inherited *DIO2* polymorphism [20,21]. Combination therapy with L-T_4_ plus L-T_3_ has been found to be helpful in some, but not all studies [22]. One review proposed that some patients likely have a compounding condition that increases the likelihood of developing symptoms [21]. Could this be elevated rT_3_ levels?

It is estimated that about 10–29% of patients with hypothyroidism use DTE as their primary thyroid hormone replacement medication in the US [23–25] despite concerns about the potential risk of thyrotoxicosis associated with DTE use [26,27]. Toleza et al. surveyed the content of online posts from three popular hypothyroidism forums from patients currently taking DTE and found the most frequently described benefits associated with DTE use were an improvement in symptoms (56%) and a change in overall well-being (34%) [25].

A 2013 crossover study by Hoang and colleagues compared levothyroxine to a DTE preparation (Armour Thyroid) [28]. They used 70 patients that were enrolled in a military healthcare system, were on a stable dose of levothyroxine, and had a normal TSH before the study started. During the study, patients lost an average of three pounds during once-a-day Armour Thyroid therapy, and at the conclusion of the study, they found that 49% (34/70) preferred Armour Thyroid, 19% (13/70) preferred levothyroxine, and 33% (23/70) had no preference. Importantly, patients had thyroid function tests performed at the beginning of the study and they compared the patients’ initial rT_3_ levels to ultimate preference for thyroid medication. The baseline (on L-T_4_) rT_3_ level was 32.3 ± 12.9 ng/dL that stayed elevated at 31.4 ± 12.1 ng/dL after receiving L-T_4_ but decreased to 21.1 ± 10.9 ng/dL (p < 0.001) following DTE treatment. rT_3_ was measured by RIA at Radim in Pomezia, Italy with the range not given. This prospective cross-over study supports that DTE lowers rT_3_ levels and is preferred by the majority of patients, although causality between rT_3_ levels and patient preference for DTE was not established and needs to be examined in larger studies.

This study was confirmed by Shakir and colleagues [29] who randomized patients to L-T_4_, L-T_4_ + L-T_3_, or DTE for 22 weeks. They found quality of life outcomes were similar among hypothyroid patients taking DTE vs L-T_4_ + L-T_3_ or L-T_4_. However, those patients that were most symptomatic on L-T_4_ preferred and responded positively to therapy with L-T_4_ + L-T_3_ or DTE. In support of our data, rT_3_ levels were highest in L-T_4_ treatment, lowest in DTE-treated patients and in the middle for those on L-T_4_ + L-T_3_ (p < 0.001). They did not find any difference between the rT3 levels in autoimmune and non-autoimmune patients and did not analyze if the rT3 levels correlated with symptoms.

The strengths of our study include a large number of subjects taking different types of thyroid preparations, similar to what many patients are taking in real world settings. Another strength is the measurement of rT_3_ levels with an accurate mass-spectrometry methodology. The limitations of the study include a potential bias of subjects more inclined to taking L-T_4_ + L-T_3_ and DTE preparations than would be seen in a typical Endocrinology clinic. However, our findings of the highest rT_3_ levels in those on L-T_4_ alone would favor higher rT_3_ levels in a typical Endocrinology clinic in which most patients are on L-T_4_ alone. Other limitations include the retrospective nature of the study, lack of objective measurements of fatigue and quality of life (QOL), and the lack of a causal relationship between rT_3_ levels and fatigue and QOL symptoms, a limitation that should be addressed in future studies.

## 5. Conclusions and future studies

In conclusion, our study found elevated rT_3_ levels in patients with symptoms of fatigue on various thyroid hormone replacements with the highest levels seen in patients on L-T_4_ replacement alone and the lowest levels seen in those on preparations that contain L-T_3_, including DTE. It would be premature to conclude that elevated rT_3_ levels are the cause of the symptoms in approximately 15% of patients on L-T_4_ replacement, and further studies are needed to understand the relationship better. Nine percent of patients not taking thyroid hormone replacement in our study had elevated rT_3_ values.

Further studies are needed to understand the implications of elevated rT_3_ values in patients both on and off thyroid hormone replacement and whether its measurement will be useful in clinical practice to guide thyroid hormone replacement. Randomized control studies are needed to determine if treatment with DTE or L-T_3_ in patients with elevated rT_3_ values will both lower elevated rT_3_ values and improve measurements of fatigue and QOL. Additional further studies are needed to determine what factors raise rT_3_ values and if correcting them improves hypothyroid symptoms. Overall, our study will open new avenues of thyroid disease research that could lead to improvement in clinical outcomes in patients with hypothyroidism.
