# Supplementary material for: Reverse T3 in patients with hypothyroidism on different thyroid hormone replacement
Source: PLoS One. 2025 Jun 9;20(6):e0325046. doi: 10.1371/journal.pone.0325046 (PMC12148341; doi:10.1371/journal.pone.0325046)
Supplement: Attachment — Submitted filename: response to reviewers.docx [file pone.0325046.s002.docx]

___________________________________________________________

Department of Internal Medicine

April 3, 2025

Apeksha Niraula, M.D.

Academic Editor

PLOS ONE

RE: PONE-D-24-59271

Reverse T3 in patients with hypothyroidism on different thyroid hormone replacement

Dear Dr. Niraula. We thank you and the reviewer for his/her helpful comments on our manuscript entitled “*Reverse T3 in patients with hypothyroidism on different thyroid hormone replacement*”. Our point-by-point response to the reviewer’s comments are below with our response in *italics*.

We have included the following items in our revised submission:

1. A rebuttal letter that responds to each point raised by the academic editor and reviewer(s) labeled 'Response to Reviewers'.

2.	A marked-up copy of our manuscript that highlights changes made to the original version labeled 'Revised Manuscript with Track Changes'.

3)	An unmarked version of our revised paper without tracked changes labeled 'Manuscript'.

# Comments to the Author

1. Is the manuscript technically sound, and do the data support the conclusions?

The manuscript must describe a technically sound piece of scientific research with data that supports the conclusions. Experiments must have been conducted rigorously, with appropriate controls, replication, and sample sizes. The conclusions must be drawn appropriately based on the data presented. Reviewer #1: Partly

*We carefully reviewed the manuscript and can state that our manuscript a technically sound piece of scientific research with data that supports the conclusions. Our experiments have been conducted rigorously, with appropriate controls, replication, and sample sizes.*

2. Has the statistical analysis been performed appropriately and rigorously? Reviewer #1: I Don't Know

*Our statistician, Martin l. Lee, PhD has reviewed the statistical portions of the manuscript and concludes that the statistical analysis been performed appropriately and rigorously.*

3. Have the authors made all data underlying the findings in their manuscript fully available? Reviewer #1: Yes

*Thank you.*

4. Is the manuscript presented in an intelligible fashion and written in standard English?

PLOS ONE does not copyedit accepted manuscripts, so the language in submitted articles must be clear, correct, and unambiguous. Any typographical or grammatical errors should be corrected at revision, so please note any specific errors here. Reviewer #1: No

*We had a professional grant writer review the manuscript and have included her corrections in the new version*

*.*

5. Review Comments to the Author

**Reviewer #1:**

1. Do not start sentence with digit and write single digit in a words. *We have changed that.*

2. Revise the abstract: Background: Check these sentence "Decades ago, endocrinologists realized that in severe illnesses" and

"Without peer-reviewed publications, these functional medicine doctors rely heavily on rT3 levels to treat patients that may have no other laboratory findings". *We have changed these sentences.*

3. Background ended with objective of the study. *We checked and the background in the abstract did not include the objective of the study*
4. Kindly elaborate the methods (In abstract): sample collection, which sample was used, which methods were used to measure fT3, fT4 and TSH. *This information has been added to the Methods section of the Abstract.*
5. Results (in abstract): Revise and mention the major findings only. *We have revised the Results section of the Abstract to only include major findings.*
6. Rewrite your conclusion (in abstract) based on the objective and findings of the study and

remove the recommendation/suggestions from conclusion section. *We have removed the last sentence of the Conclusions section.*

7. The first paragraph of the result section includes the subgroup of the study population. It is better to mention it in the materials and methods. *We have moved the subgroup of the study population to the Methods section.*
8. Kindly mentioned the name statistical tests applied in the result section to test the significance. *The statistical tests are described in the* ***2.2 Statistical Analyses.*** *We have moved the mention of the Fisher’s exact test to the Methods section.*
9. Kindly revise the result section with appropriate table and figure. *We have removed Table 2 and renumbered the remaining table numbers*. Are same data presented in table 2 and figure 1? *We agree that Table 2 and Figure 1 are duplications and have removed Table 2.*

10. Write the conclusion in a separate section. *We have added a* ***Conclusions and Future Studies*** *Section.*

We look forward to hearing from you after the re-review process.

Sincerely yours,

Theodore C. Friedman, M.D., Ph.D.

Chairman, Department of Internal Medicine

Chief, Division of Endocrinology, Metabolism and Molecular Medicine

Professor of Medicine-Charles R. Drew University of Medicine & Science

1731 E. 120th St. Los Angeles, CA 90059

Professor of Medicine UCLA

[theodorefriedman@cdrewu.edu](mailto:theodorefriedman@cdrewu.edu)

phone (323) 539-8020

fax (310) 564-2786
